# Job Satisfaction and Turnover Intention Among People With Disabilities Working in Special Employment Centers: The Moderation Effect of Organizational Commitment

**DOI:** 10.3389/fpsyg.2020.01035

**Published:** 2020-06-03

**Authors:** Marina Romeo, Montserrat Yepes-Baldó, Claudia Lins

**Affiliations:** Social Psychology and Quantitative Psychology Department, Faculty of Psychology, Universitat de Barcelona, Barcelona, Spain

**Keywords:** job satisfaction, organizational commitment, turnover intention, employees with disabilities, Special Employment Centers

## Abstract

With the goal of contributing to the growth of research on people with disabilities in employment, in particular in relation to their job satisfaction (JS), organizational commitment (OC), and turnover intention (TI), this study explores the effect of JS on TI among employees with disabilities and the moderation effect of OC and its four dimensions on this main relationship. A total of 245 Special Employment Center (SEC) employees in Spain answered a questionnaire. To analyze the results, a descriptive analysis with bivariate correlations across the variables was performed, and the moderation model was tested subsequently using macro PROCESS for SPSS by Hayes. For the significant effects, a pick-a-point approximation was used to interpret the results. The results show that OC and its dimensions have no significant effect on the direct relationship. However, some components of JS, such as the relationship with co-workers and with supervisors, play a significant role in the relationship with TI when moderated by affective and value commitment. Our results show that it is important that human resources departments create conditions favoring a work environment with positive interpersonal relationships between employees and managers in order to minimize TI at SECs.

## Introduction

According to the International Labor Organization (International Labour Organization, 2019), the world population includes an estimated one billion people with disabilities (15%), of whom 785 million (about 80%) are of working age.

In Spain, as stated by the Spanish State Public Employment (SEPE) Service in its Labor Market Report on People with Disabilities (SEPE, 2019), there are 1,860,600 people of working age with disabilities, representing 6.2% of the total working age population in the country.

In the global push to promote inclusion of people with disabilities, the International Labour Organization (2019) highlighted that their access to employment not only is a right but also offers economic benefits, given that the inclusion of people with disabilities in the labor market could increase the GDP of developing countries by up to 7%.

Some countries have introduced clear laws and incentives to promote inclusion. One example of a European country that has done so is Spain. Spanish law determines that people with disabilities have the right to work in conditions that guarantee the application of the principles of equal treatment and non-discrimination (Royal Legislative Decree 1/2013, 2013 art. 35). To incentivize employment, the legislation also establishes that in public and private companies with 50 or more employees, at least 2% must be people with disabilities (Royal Legislative Decree 1/2013, 2013 art. 42).

By way of a contribution to the growth of studies that take into account people with disabilities as employees, this research aimed to study how their turnover intention (TI) is affected by job satisfaction (JS) with regard to salary, physical conditions, work stability, the relationship with co-workers and supervisors, recognition by supervisors, professional development opportunities, and social benefits.

Attracting, engaging, and retaining a diverse workforce are a priority for organizations today. Therefore, understanding the factors that are associated with the satisfaction of disabled employees is important, given that disabled individuals constitute an underused labor pool and can offer organizations the benefits of diversity (Shantz et al., 2018).

Complementing this idea, Baumgärtner et al. (2015) affirm that understanding why and how the JS of various groups of employees differs and what can be done to raise the satisfaction levels of these groups is important for organizations interested in the long-term inclusion of all employees and their financial success.

Several studies have also linked JS to organizational commitment (OC; Iverson and Roy, 1994; Lok and Crawford, 2001; Del Junco and dos Santos, 2008; Ahmad et al., 2019). Specifically, Del Junco and dos Santos (2008) mention that the relationship between OC and JS is particularly important for organizations, since employees who show a commitment to the organization and are satisfied with their work usually offer a guarantee of better performance, thereby minimizing absenteeism, turnover, and TI.

In this context, our research aimed to analyze the moderation effect of OC (Quijano et al., 2000) on the relationship between JS (Vroom, 1964; Harpaz, 1983; Yepes-Baldó, 2010) and TI (Tnay et al., 2013) among people with disabilities working in Special Employment Centers (SECs) in Spain.

The Human System Audit (HSA) was the theoretical approach used to understand the interplay between these concepts within a broader integrative model. It is an “integrated proposal for intangible assessment, quality assessment in excellence models and, in general, for diagnosis and intervention in the human system in organizations, as well as for investigating organizational human behavior” (Quijano et al., 2008, 92).

Additionally, the context of our research was provided by the SECs. These are defined as organizations where the inclusion of people with disabilities (Royal Decree 1/20132273/1985, 1985 art. 42) is a stated social objective. According to this royal decree, these centers may be opened directly by the public administrations or by natural or legal persons who meet the appropriate civil requirements. They can be public or private, profit or non-profit organizations (Royal Decree 1/20132273/1985, 1985 art. 5). The law also stipulates that at least 80% of the people employed by a SEC must have a legally recognized disability of at least 33% (mental or intellectual) or 65% (physical or sensory).

## Theoretical Framework and Hypotheses

As we have already pointed out, the main theoretical framework used in this research was the HSA model. Specifically, the definition of JS was established from this perspective. It is based on Vroom (1964) and Harpaz (1983) and defined as “a generalized attitude toward work, which includes feelings and affective responses, evaluative appraisals, and predispositions toward certain behaviors” (Yepes-Baldó, 2010, p. 103). JS is related to certain aspects of work and the employing organization, such as salary, physical conditions, job stability, relationships with co-workers, relationships with supervisors, recognition by supervisors, professional development opportunities, and social benefits (Locke, 1976; Meliá et al., 1986; Yepes-Baldó, 2010).

Despite the many studies addressing JS, not much research has explored the JS of people with disabilities (Houser and Chace, 1993; Rodríguez et al., 2014; Baumgärtner et al., 2015; Pagán-Rodríguez, 2015; Park et al., 2016; Akkerman et al., 2017, 2018; Kocman and Weber, 2018; Shantz et al., 2018).

Akkerman et al. (2016) affirm that little is known about the JS of employees with disabilities, but given the enormous significance of work in people’s lives, awareness of their JS is essential. They also mention that it has been suggested that people with disabilities are satisfied with their jobs for much the same reasons as non-disabled employees (Moseley, 1988; Goode, 1989; Parent et al., 1996). As Moseley (1988) points out: “workers with severe disabilities and their non-disabled coworkers are more alike than different. The aspects of a job that are important to one are important to the other” (p. 217).

### Job Satisfaction and Turnover Intention

Job satisfaction is understood by the HSA as an individual-level result, reflecting the quality of human resources. Therefore, it is “an antecedent of important organizational outcomes, such as personnel TI” (Romeo et al., 2011b, p. 901). TI can be defined as “conscious and deliberated willfulness of an individual toward voluntary, permanent withdrawal from the organization” (Tett and Meyer, 1993, p. 262). Unlike actual turnover, turnover intent is not explicit, and intentions can be understood as a statement about a specific behavior of interest (Berndt, 1981). Since it is an immediate precursor to actual turnover, an employee’s decision to quit an organization is an undesirable outcome for the organization and the employee as it affects both of them in many ways. That is why it is considered very important to understand its predictors in order to minimize its negative impact on the performance of an organization performance (Low et al., 2001).

When analyzing the relationship between JS and TI among employees with disabilities, Pérez et al. (2015) found that there is a significant negative relationship between JS and TI. Schur et al. (2017) also presented the same results but complemented them by showing that employees with disability presented lower JS in comparison with non-disabled workers.

H_1_. Job satisfaction (and its components) among employees with disabilities are negatively related to their turnover intention.

### Organizational Commitment as a Moderator

Employee JS and OC have been negatively related to actual turnover and intention to leave (Arnold and Feldman, 1982; Bluedorn, 1982; Hollenbeck and Williams, 1986; Tett and Meyer, 1993), and positively among them (Bluedorn, 1982; Clegg, 1983; Dougherty et al., 1985). Although JS correlates more strongly than commitment to TI, they both make a unique contribution to it (Tett and Meyer, 1993).

There are few studies that have specifically analyzed the relationship between JS, commitment, and TI among employees with disabilities. Schur et al. (2017) found that in comparison with non-disable workers, employees with disability presented similar levels of OC and TI, but lower levels of JS.

Different studies have analyzed the effect of commitment as a moderator on different pairs of relationships: incivility and well-being (Kabat-Farr et al., 2018); job demands and well-being (Rivkin et al., 2015); transformational leadership and psychological strain (Franke and Felfe, 2011); stress and performance (Jamal, 2011); and work stressors and JS (Lu et al., 2010). In all these cases, commitment protected against negative relations and, for this reason, we hypothesized that the employees committed to the organization and satisfied with their work usually offer a guarantee of better performance, thereby minimizing absenteeism, turnover, and TI (Del Junco and dos Santos, 2008). These results suggest an interaction effect between both variables when explaining TI.

H_2_. The organizational commitment of employees with disabilities moderates the relationship between job satisfaction (and its components) and turnover intention.

Furthermore, some other studies have examined the roles played by employee JS and OC as predictors of TI, but apparently none of them approached these antecedents from a multidimensional perspective on these antecedents (Tett and Meyer, 1993; Chen, 2006; Vandenberghe and Tremblay, 2008; De Gieter et al., 2011; Tnay et al., 2013; Larkin, 2015).

In this sense, this research adopted a multidimensional perspective to examine commitment, developed within the framework of the HSA model (Quijano et al., 2000). From this perspective, OC is defined as “a psychological link that employees build up with the organization for different reasons” (p. 34). The integrated model of OC has four different dimensions: needs, exchange, affective commitment, and values commitment (Romeo et al., 2011a, b).

Need commitment is focused solely on keeping a job as a way to survive; exchange commitment is based on the balance between remuneration/rewards received and input; affective commitment refers to the emotional bond derived from the need for attachment; and values commitment is related to the recognition of common goals and values shared by the employees and their employers (Romeo et al., 2011a, b).

As explained above, commitment interacts with JS to reduce TI. In this regard, we hypothesized that the negative relationship between JS and TI is only possible with high levels of exchange, affective and values commitment, and low levels of needs commitment. In this last case, low levels of satisfaction and high levels of needs commitment should increase the probability of TI.

H_2a_. The need commitment of employees with disabilities moderates the relation between job satisfaction (and its components) and turnover intention.
H_2b_. The exchange commitment of employees with disabilities moderates the relation between job satisfaction (and its components) and turnover intention.
H_2c_. The affective commitment of employees with disabilities moderates the relation between job satisfaction (and its components) and turnover intention.
H_2d_. The values commitment of employees with disabilities moderates the relation between job satisfaction (and its components) and turnover intention.

## Methods

### Design and Procedure

This study presents a cross-sectional design with a protocol characterized as self-reported measure. First, the researchers contacted the managers of the centers and invited them to participate. Following acceptance, direct support employees administered the survey to the participants. A cover letter was included with information about the purpose of the survey, the research ethics protocols, and the survey itself. To facilitate participation and help employees with special needs, the survey was administered in groups in some centers, in a room with computers. For those who needed additional help, the direct support employees provided standardized clarifications. Participation in the survey was voluntary and strictly confidential.

### Participants

Employees with different types of disabilities at various SECs in Spain were invited to participate in the research. Following prior contact, 350 employees from all over Spain were invited to answer the questionnaire. Participation was voluntary, and 245 people finally participated in the study by completing the questionnaire, which represents a response rate of 70%.

Some socio-demographic variables were collected, corresponding to gender, age, tenure, previous occupation, type of contract, position held, educational level, work schedule, and type and degree of disability.

The average age of the participants was 41.5 years (SD = 9.56; range = 19–64). The majority were women (55%), working full-time (66.1%) in production tasks (56.8%), with an indefinite contract (54.7%), and studies completed at secondary (28.2%) or primary (23.5%) education level. The average tenure was 6.39 years (SD = 6.96; range = 1–27). 40% of the participants were unemployed before being hired by the SEC, and 27.1% came from the ordinary labor market. Regarding the type of disability, most participants had a physical (36.7%) or intellectual disability (29%), and their average degree of disability was 44.29% (SD = 12.81; range: 33–82%).

### Instruments

#### Satisfaction

The HSA-SAT questionnaire was administered. This measures employee JS according to the evaluation of the specific levels of eight distinct aspects based on a single-item scale: salary, physical conditions, job stability, relationships with co-workers, relationships with supervisors, recognition by supervisors, professional development opportunities, and social benefits. One example of an item from the questionnaire was “I am satisfied with my salary.” The questionnaire presented eight items and a five-point Likert scale ranging from 1 (very dissatisfied) to 5 (very satisfied). The previous Cronbach’s alpha was 0.90 (Yepes-Baldó, 2010).

#### Organizational Commitment

The validated Identification-Commitment Inventory (ICI) questionnaire developed by Romeo et al. (2011a) was used to measure OC. It distinguishes four dimensions of commitment: needs commitment, exchange commitment, affective commitment, and values commitment. Some examples of questions are “I would not recommend working in this organization to any family member or friend” (needs commitment); “I continue working in this organization because I consider the advantages (social benefits, work schedule, salary, etc.) I obtain to be fair” (exchange commitment); “I feel emotionally attached to my organization” (affective commitment); and “I feel that there is a great similarity between my personal values and those of my organization” (values commitment). The questionnaire presented eight items and a five-point Likert scale ranging from 1 (strongly disagree) to 5 (strongly agree). The Cronbach’s alpha was 0.87 for values commitment, 0.88 for affective commitment, 0.89 for exchange commitment, and 0.91 for needs commitment (Romeo et al., 2011a).

#### Turnover Intention

Turnover intention was measured with an *ad hoc* single item (“I am going to look for another job next year”). It used a five-point Likert scale ranging from 1 (strongly disagree) to 5 (strongly agree).

### Data Analysis

The data analysis consisted of descriptive analysis, Spearman’s correlation matrices to test bivariate relations across all variables, and the use of the PROCESS macro created by Hayes (2013) to test the moderated models. Subsequently, based on the pick-a-point approximation, graphical computational tools were also used to further explore the interactions of the significant predictor’s items and moderator variables (Hayes, 2013). The internal consistency of the scales, measured by Cronbach’s alpha, was also calculated.

Additionally, Harman’s single-factor test (Podsakoff et al., 2003) was used to analyze whether there was any presence of common method bias in our data, since we were only using questionnaires to collect data. The principal component analysis of all the variables produced five distinct factors. Four of the factors accounted for 64.18% of the total variance, but the other factor accounted for more than 50% of the variance (32.89%). However, this factor included all the items related to satisfaction. Thus, it can be affirmed that common method bias was not a serious threat.

## Results

### Descriptive and Bivariate Correlations Analysis

The descriptive analyses are presented in Table 1. Results show that general commitment and affective and values commitment had the highest levels (on a five-point scale). On the other hand, needs commitment and TI had the lowest levels.

**TABLE 1:** Descriptive analysis, Spearman’s correlation coefficients, and Cronbach’s alpha.

Variables	N	Mean	SD	1	2	3	3.1	3.2	3.3	3.4
1. Job satisfaction	245	3.30	0.85	(0.78)						
2. Turnover intention	238	2.88	1.02	–0.11	(–)					
3. Commitment	242	3.50	0.65	0.47**	−0.43**	(0.78)				
3.1 Need	242	2.35	0.91	−0.32**	0.10	−0.60**	(0.44)			
3.2 Exchange	242	3.26	0.93	0.53**	−0.17**	0.77**	−0.20**	(0.75)		
3.3 Affective	242	3.66	0.82	0.47**	–0.06	0.86**	−0.38**	0.63**	(0.67)	
3.4 Values	241	3.44	0.83	0.50**	–0.09	0.75**	−0.29**	0.48**	0.59**	(0.60)

Cronbach’s alpha in parentheses; **p < 0.001.

Table 1 shows the results of the bivariate correlation analysis. Contrary to our expectations, JS did not correlate with TI. Nevertheless, TI correlated with some of the components of JS. Specifically, it correlated negatively (*p* < 0.01) with salary satisfaction (*r* = −0.231; adjusted *R*^2^ = 0.048; *b* = −0.228), physical conditions (*r* = −0.200; adjusted *R*^2^ = 0.036; *b* = −0.201), job stability (*r* = −0.227; adjusted *R*^2^ = 0.046; *b* = −0.224), relationship with supervisors (*r* = −0.256; adjusted *R*^2^ = 0.068; *b* = −0.268), recognition by supervisors (*r* = −0.253; adjusted *R*^2^ = 0.057; *b* = −0.248), and professional development opportunities (*r* = −0.223; adjusted *R*^2^ = 0.033; *b* = −0.194). The level of satisfaction as regards social benefits and the relationship with co-workers bore no relation to TI. Regression analyses confirmed these relations. These results partially support H_1_.

Additionally, JS had a positive correlation with general commitment and exchange, affective and values commitment, and a negative one with needs commitment. Finally, general commitment and exchange commitment correlated negatively with TI. There was no correlation between TI and needs, affective, and values commitment.

### Moderation Effect of Commitment on the Relationship Between Job Satisfaction and Turnover Intention

**FIGURE 1:**
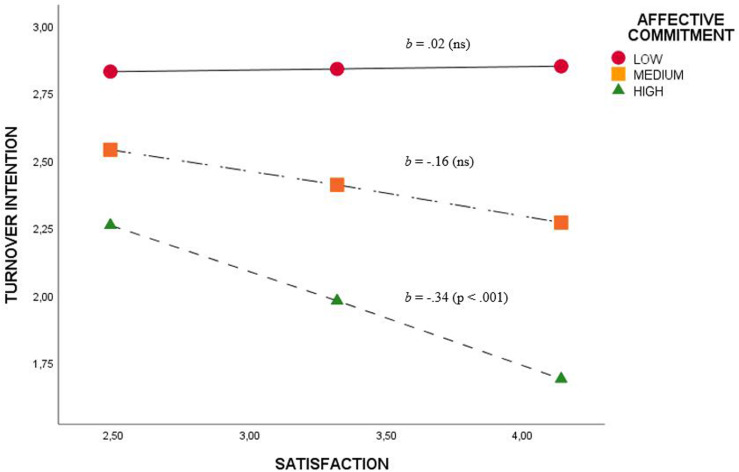
Moderation effect of affective commitment on the relationship between satisfaction and turnover intention.

After performing the Hayes PROCESS macro regression to test the moderation effects (H_2_, H_2__a_, H_2__b_, H_2__c_, and H_2__d_), it was possible to confirm that only affective commitment moderates the relationship between JS and TI (H_2__c_) (*b* = −0.22; *p* < 0.03). In this sense, JS, with high levels of affective commitment, had a negative effect on TI (Figure 1).

Moderation models were tested to analyze the results in greater detail, considering each one of the eight JS components in relation to general commitment and its four dimensions.

As regards general, needs, and exchange commitment, the results showed no interaction effects in any case. In the case of affective commitment, two interaction effects were found in the relation between TI and satisfaction with the relationship with co-workers (b = −0.23; *p* < 0.001) and supervisors (*b* = −0.21; *p* < 0.001).

**FIGURE 2:**
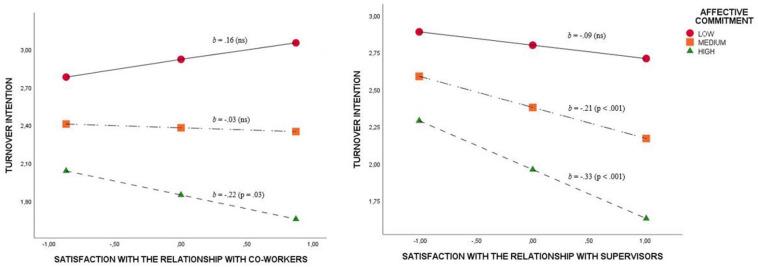
Moderation effect of affective commitment on the relation between turnover intention and satisfaction with the relationship with co-workers and supervisors.

As shown in Figure 2, concerning high levels of affective commitment, satisfaction with the relationship with co-workers had a minimizing effect on TI. Likewise, with medium and high levels of affective commitment, satisfaction with the relationship with supervisors also had a negative effect on TI (Figure 2).

**FIGURE 3:**
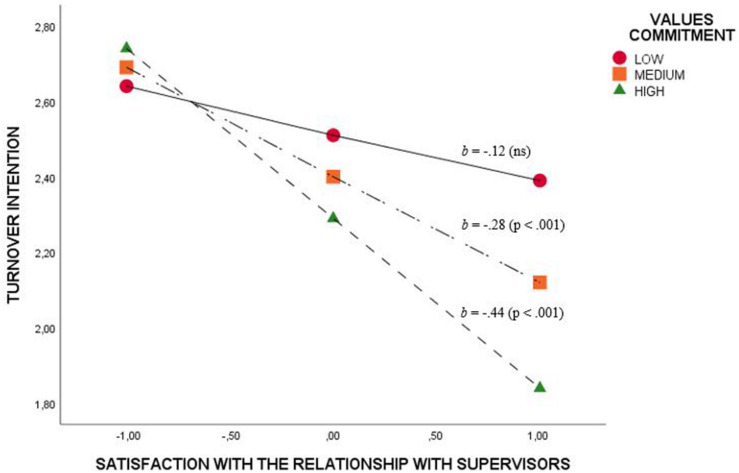
Moderation effect of values commitment on the relation between turnover intention and satisfaction with the relationship with supervisors.

Finally, regarding values commitment, an interaction effect was found in the relation between TI and satisfaction with the relationship with supervisors (*b* = −0.19; *p* = 0.02). In this sense, with medium and high levels of values commitment, satisfaction with the relationship with supervisors had a minimizing effect on TI (Figure 3).

## Discussion and Conclusion

Employees with disabilities at SECs showed medium–high levels of affective and values commitment to their organizations, medium levels of general satisfaction, and low levels of needs commitment and TI.

As regards the different components of satisfaction, employees with disabilities showed medium–high levels of satisfaction with their supervisors and colleagues. Similar results were obtained in a previous study of SECs in France (Fernández de Soto, 2017). It is important to take into account that our results may be explained by the context of a sheltered work environment. Conversely, Schur et al. (2017) found that employees with disabilities who worked in ordinary companies perceive worse employee-management relations and worse treatment by management, although they report similar co-worker relations as employees without disabilities (Schur et al., 2017).

The results partially confirmed our hypotheses from a general perspective. First, as regards the relationship between satisfaction and TI (H_1_), our results are similar to those obtained by Tett and Meyer (1993) for people without disabilities. They found that greater employee JS is invariably reported as negatively related to turnover and intentions to leave. Our results confirm that this connection is significant except in the case of satisfaction with social benefits and the relationship with co-workers with respect to TI.

Second, regarding the moderation effect of OC (H_2_), only affective commitment was found to moderate the relationship between general satisfaction and TI (H_2__c_). The results of the detailed analysis of JS components, and each of the four dimensions of OC showed that three interactions were significant. Affective commitment moderates the relationship between TI and satisfaction with co-workers and supervisors, while values commitment moderates the relationship between and TI and satisfaction with the relationship with supervisors.

In view of the results, it may be affirmed that when there is a high level of affective commitment, satisfaction with co-workers will lead to lower TI. Regarding the second significant interaction, it can be interpreted that when there are high or medium levels of affective commitment, satisfaction with supervisors will also lead to lower TI. Regarding the third interaction, with average and high levels of value commitment, satisfaction with supervisors will result in lower TI. Our results concur with Schur et al. (2017), who affirmed that, “perceptions of better treatment by management and coworkers predict lower TI” (p. 488).

These findings can help HR departments to develop strategies to attract, engage, and retain these employees, who are a priority for organizations today, given that disabled individuals constitute an underused labor pool and can offer organizations the benefits of diversity (Shantz et al., 2018).

In this context, it is important for organizations to promote an organizational culture oriented to people’s needs (Cook and Lafferty, 1986) in order to assure high levels of JS—especially with co-workers and supervisors—and, consequently, lower levels of TI. This implies that the members of organizations, managers, and employees should focus their efforts on guaranteeing positive interpersonal relationships. In this sense, it is important to have a friendly, open, and sensitive attitude to work group satisfaction, stimulating personal and professional development. All this ensures a positive work experience, which has been related to the development of affective commitment (Mowday et al., 2013).

### Limitations and Future Research

Some limitations should be taken into account. First, almost 30% of the sample had intellectual disabilities. Although they received support when requested, some of them may not have fully understood the questions, and this may have affected the results of the study.

Furthermore, it is important to point out that this collective encounters greater difficulties when accessing the ordinary market and that SECs provide them with a sheltered employment opportunity. Additionally, our sample is representative of the general population of employees at SECs, as it has a similar distribution of employees according to their disability: 42% of employees with psychological problems in SECs (including intellectual disability and mental disorders), 48% with physical disabilities, and 10% with sensorial disabilities (KPMG, 2011).

Second, to keep the questionnaire short and ensure it was easily understood by the participants, the variable TI had only one item, with a single scale. Although Fisher et al. (2016) have demonstrated the adequateness of the use of single items to measure TI in organizational and occupational psychology, adding new items or validated scales might well enrich the results.

Third, with regard to the instruments, needs commitment presented a value of Cronbach’s alpha of 0.44, which is considered unacceptable (Cronbach, 1951). One detail that may help to explain this low value is that one of the answers on the need commitment scale was as follows: “I would not recommend working in this organization to any family member or friend.” Bearing in mind that SECs are organizations that exclusively employ people with disabilities, it seems logical that they would not recommend them to other family members and friends if they do not have disabilities. This suggests a need to rephrase the question for future research.

Fourth, given that Hartman’s single test indicated that common method bias could be a relative problem in this study, future research should include other methods such as open interviews and observation, as well as the application of self-evaluation questionnaires, thereby avoiding the risk of generating spurious correlations through common-method variance (Podsakoff and Organ, 1986).

Finally, future research could use larger samples and an analysis of employees with disabilities who work in ordinary companies would bring in additional results that could complement the research carried out on this sector of the population.

## Data Availability Statement

The datasets generated for this study are available on request to the corresponding author.

## Ethics Statement

Ethical review and approval was not required for the study on human participants in accordance with the local legislation and institutional requirements. The patients/participants provided their written informed consent to participate in this study.

## Author Contributions

MR and MY-B contributed to the theory development, research design, data analyses, and discussion. CL contributed to the theory development and data analyses.

## Conflict of Interest

The authors declare that the research was conducted in the absence of any commercial or financial relationships that could be construed as a potential conflict of interest.
